# Effects of fermented feed on growth performance, nutrient metabolism and cecal microflora of broilers

**DOI:** 10.5713/ab.21.0333

**Published:** 2021-10-29

**Authors:** Jiantao Li, Lijuan Tao, Rong Zhang, Guiqin Yang

**Affiliations:** 1 College of Animal Science and Veterinary Medicine, Shenyang Agricultural University, Shenyang, Liaoning Province, 110866, China

**Keywords:** Broiler, Fermented Feed, Growth Performance, Microflora, Nutrient Metabolism

## Abstract

**Objective:**

To investigate the effects of enzyme-bacteria co-fermented feed on broilers, the basal diet (BF) was pretreated by microbial enzyme co-fermentation, and then different proportions of BF were replaced to study its effects on growth performance, nutrient metabolism and cecal microflora of broilers.

**Methods:**

Four hundred and eighty 1-day-old broilers were randomly divided into 6 groups. The control group was fed with BF, and groups 1 to 4 were treated with dried fermented feed (DFF) instead of 10%, 15%, 20%, and 25% the BF, and group 5 was treated with wet fermented feed (WFF) instead of 10% the BF, named BF, 10% DFF, 15% DFF, 20% DFF, 25% DFF, and 10% WFF, respectively. The trial period was 42 days.

**Results:**

The results showed that the average daily feed intake and average daily gain of 10% DFF, 15% DFF, and 10% WFF groups were significantly higher than those of the control group at 22 to 42 days and 1 to 42 days (p<0.05). Except for 10% DFF group, *Firmicutes* of all treatment were higher than that of control group. The *Bacteroides* of each treatment group were lower than that of the control group (p>0.05). At the same time, the nutrient apparent metabolic rate and cecal microbial abundance of each treatment group had an increasing trend (p>0.05).

**Conclusion:**

In conclusion, the feed fermented by enzyme and bacteria had a potential promoting effect on the growth performance and nutrient digestibility of broilers.

## INTRODUCTION

With the increasing scarcity of feed resources and the urge for reduced use of antibiotics, it has become the priority target to explore diversified raw materials and efficient production modes. It can be acknowledged that fermented feed has been favored in animal husbandry for its ease of production. Fermented feed refers to the degradation of macromolecular substances in feed into small molecules through microbial metabolism under manual control, to improve the digestibility of macro and micronutrients [1]. Fermentation can decompose or transform the antinutritional factors into non-toxic components, thus reducing the content of antinutritional factors and toxic compounds [2]. Fermentation has gradually become one of the important methods for detoxification of feed mycotoxins. In addition, the positive effects of microorganisms and their metabolites in fermented feed on intestinal health have been confirmed by many researchers. Studies have shown that fermented feed is rich in probiotics and their metabolites, which can improve the intestinal microecological balance and animal immunity [3]. Zhao et al [4] found that adding different proportions of fermented feed to the diet of layers improved the quality of eggs. Also, the conclusion that adding 10% fermented feed can improve the intestinal microecological balance and reduce the excretion rate of nitrogen and phosphorus was also confirmed by Zhao et al [5]. Moreover, the technology of fermented feed preparation by bacteria and enzymes has been gradually recognized by reseachers. Sun et al [6] co-fermented cottonseed meal with *Bacillus* and papain, which significantly reduced the content of crude fat, crude fiber and free gossypol in cottonseed meal. Another result showed that the content of free gossypol and glucosinolates in the miscellaneous meal treated with *Saccharomyces cerevisiae*, *Lactobacillus* and cellulase decreased significantly, while the content of crude protein (CP), small peptides and amino acids increased [7]. Although the positive role of fermented feed in livestock breeding has been recognized by many researchers, the application effect of fermented feed prepared under different production processes is not very consistent [8–10]. In addition, the mechanism of the effect of fermented feed on intestinal microecology needs to be further explored [11]. So, to further investigate the effect of fermented feed on broilers, we pretreated the basal diet by microbial enzyme co-fermentation, and then changed the basal diet in different proportions to study its effects on growth performance, nutrient metabolism and cecal microflora of broilers.

## MATERIALS AND METHODS

### Experimental design and management of birds

The management and design of the experiment were kept to animal care rules approved by the Institutional Animal Care and Use Committee of Shenyang Agricultural University (202006046).

A total of 480 1 day-old Arbor Acre (AA^+^) broilers were randomly assigned to 6 treatments, each with 8 replicates (10 chicks per replicate). The control group was fed with basal diet (BF) (Table 1), and groups 1 to 4 were treated with dried fermented feed (DFF) instead of 10%, 15%, 20%, and 25% BF, and groups 5 was treated with wet fermented feed (WFF) instead of 10% BF, and were named BF, 10% DFF, 15% DFF, 20% DFF, 25% DFF, and 10% WFF, respectively.

Birds were reared in multi-tiered brooder cages and raised in climate-controlled rooms at the Shenyang Agricultural University, China. Birds had *ad libitum* access to feed and water over the trial period. The initial brooding temperature was 35°C; this was gradually reduced to 21°C at 35 days of age and fixed at this level until the end of the experiment. Twenty-four hours of lighting were provided uninterrupted every day.

### Preparation of fermented feed

First, corn, soybean meal, corn gluten meal, dried distillers grains with solubles (DDGS) and wheat bran were prepared into the air dried feed to be fermented according to the proportion provided by the basal diet (Table 2). Then the lactic acid bacteria were inoculated in MRS (De Man, Rogosa, and Sharp) liquid medium at 2% and incubated at 35°C for 12 h to prepare starter culture. The starter culture was inoculated into the air dried feed to be fermented, and the moisture content was adjusted to 30%. The fermented feed was put into a breathing bag with a one-way breathing valve (only exhaust but not intake). The temperature was 32°C for anaerobic fermentation for 5 days. The pH was recorded before and after fermentation.

### Growth performance

The birds were weighed at 1 d, 21 d, and 42 d, the leftover feed was recorded every day to measure the average daily feed intake (ADFI), average daily gain (ADG), and ADFI/ADG (F/G). ADFI, ADG, and F/G data were corrected for mortality.

### Apparent metabolic rate

The total fecal collection method was used to test the metabolizable energy and nutrient metabolism rate of feed in 38 to 42 days. Each morning, all the remaining feed in the trough was recovered, and the actual daily feed intake of each replicate chicken was accurately recorded. At 8 am, a scraping board was used to collect all the excrement on the cardboard, and the data were recorded. The feathers, feed and dander mixed in the excreta were picked up. The excreta were collected for 4 days continuously. All excreta collected for 4 days were dried in 65°C oven to constant weight and weighed after 24 hours to determine moisture loss. Then smashed, passed through a 40 mesh sieve (mesh diameter is 0.45 mm), mixed well. The method of total energy and chemical components were determined according to the method of Zhang [12].

### Sample collection

At the end of the experiment, six chickens in each treatment were randomly selected and killed by venous bloodletting. The cecum was dissected, and the contents were extruded into a cryopreservation tube and stored at −80°C. Then, the 16S rRNA microbial sequencing of cecal contents was conducted.

### The determination of cecal microflora

The extraction and concentration detection of DNA were carried out according to Li [13]. Then prepare polymerase chain reaction (PCR) amplification reaction system in sterile PCR tube (Table 3). The reaction procedure for PCR was showed in Table 4. During the second PCR amplification, the reaction system and reaction procedure are shown in Table 5 and 6, respectively. At the end of the PCR, 1% agarose gel electrophoresis was carried out. The electrophoresis voltage was 130 V and the time was 20 min. Photographs were taken in the UV Gel imaging system and preserved. DNA purification, recovery and quantification. Bioinformatics analysis of cecal microflora: after the samples were processed, Shenggong Bioengineering Co., Ltd. (Shanghai, China) was entrusted to conduct high-throughput sequencing.

### Statistical analysis

Data were analyzed by one-way analysis of variance using SAS 9.2. Comparisons of means for treatments were made using Duncan’s multiple range tests. Significance was accepted at p<0.05. In addition, the data of microbial sequencing are counted on the platform provided by Shenggong Bioengineering Co., Ltd. (China).

## RESULTS

### Comparison of nutritional components of feed before and after fermentation

The comparison of nutrients before and after fermentation is shown in Table 7. After fermentation, the total acid, and the number of *Lactobacillus* increased significantly (p<0.05). However, pH decreased significantly (p<0.05). Compared with the fermented feed, the total acid content, and the number of *Lactobacillus* in the feed dried at 35°C were significantly (p<0.05) higher than those in the feed not dried. After fermentation, the moisture was reduced to the same level as that before fermentation, and no significant (p>0.05) changes were found in other general nutritional indexes.

### Growth performance

As shown in Table 8, at 22 to 42 days of age, ADFI and ADG of broilers in the 10% DFF, 15% DFF, and 10% WFF groups were significantly higher than those in the control group (p<0.05). At 1 to 42 days of age, compared with the control group, the ADFI and ADG of broilers in the 10% DFF, 15% DFF, and 10% WFF groups were significantly increased (p< 0.05). Fermented feed (after drying) can replace 10% to 15% of basal diet, but the growth performance of broilers cannot be further improved by increasing the proportion of fermented feed.

### Apparent metabolic rate

The nutrient metabolic rate of broilers has a trend of improvement by adding fermented feed (Table 9). Compared with the control group, the apparent digestibility of CP, ether extract (EE), calcium and phosphorus in the diet of broilers were increased but did not reach a significant level (p>0.05).

### Cecal microflora

After 16S rRNA sequencing, 19 phyla were detected (Table 10), and 6 phyla with relative abundance greater than 0.05% were *Firmicutes* (68.06%), *Bacteroidetes* (24.92%), *Proteobacteria* (4.65%), *Synergistetes* (1.03%), *Verrucomicrobia* (0.16%), *Euryarchaeota* (0.09%), unclassified (0.85%), others (0.24%). *Firmicum* and *Bacteroides* were the dominant flora. Except for 10% DFF group, *Firmicum* of all treatment groups were higher than that of control group. The *Bacteroides* of each treatment group were lower than that of the control group, but the difference was not significant (p>0.05).

At genus level, 14 genera with relative abundance greater than 1% were detected (Table 11). They were: *Alistipes* (16.64%), *Luminococcus* (4.79%), *Lactobacillus* (4.33%), *Faecalibacterium* (4.26%), *Faecalibacterium* (3.22%), *Subdoligranulum* (2.77%), *Romboutsia* (2.13%), *Barnesiella* (1.97%), *Clostridium xlva* (1.82%), *Clostridium IV* (1.51%), *Bilophila* (1.48%), *Butyricoccus* (1.31%), *Vampirovibrio* (1.14%), *Dorea* (1.06%), Unclassified (41.25%). The proportion of *Alistipes*, *Ruminococcus*, *Lactobacillus* and *Bacteroides* in cecum of broilers were changed by adding fermented feed, but only *Ruminococcus* was significantly affected (p<0.05).

### Microbial diversity

The abundance and diversity of cecal microflora can be reflected by alpha diversity analysis (Table 12). The sequencing coverage of each group of samples is more than 98.6%, and the depth is enough to reflect the microflora in the samples. Adding fermented feed could increase the Chao 1 and ACE index of cecal microflora in broilers, but it did not reach a significant level (p>0.05).

## DISCUSSION

### Growth performance

Fermented feed can degrade macromolecular and antinutritional factors in raw materials under the action of microorganisms, thereby increasing feed digestibility and absorption, resulting in improved growth performance of broilers [14,15]. The effect of fermentation on nutrient digestibility and utilization of feed has been confirmed in mink and salmon [16,17]. Microorganisms and their metabolites can improve the intestinal microecological environment, enhance the resistance to diseases, and contribute to the maintenance of intestinal health, which is an effective alternative strategy for antibiotics. Using the synergistic effect of microorganisms and enzymes to predigest the feed can make the degradation of macromolecular substances in the feed more thorough, the microbial fermentation efficiency is higher, and the effect on broilers is better than single fermentation or enzymatic hydrolysis [18].

Growth performance is an important index of feed fermentation quality. Chen et al [19] fed broilers with different proportions of fermented feed (*Lactobacillus* and *Bacillus subtilis* as fermentation strains) instead of complete formula feed. The results showed that when the proportion of fermented feed was 10%, ADG of broilers could be increased and F/G could be reduced. Li et al [20] showed that adding 10% and 15% fermented complete formula feed to broiler diet could improve ADG and reduce F/G. The results showed that adding 10% and 15% fermented feed could increase ADFI and ADG and reduce F/G of broilers aged 22 to 42 d and 1 to 42 d, which was consistent with the previous reports. However, at the age of 1 to 21 d, the F/G of broilers with 25% fermented material or 10% wet fermented material increased. The results showed that the digestibility of organic matter in ruminants and monogastric animals decreased by 0.65% to 0.70% and 1.35% to 1.40% when the level of crude fiber in the diet increased by 1% [21]. Too much insoluble fiber will shorten the residence time of chyme in the intestine, and too much soluble fiber will adhere to the surface of chyme to form a nutritional barrier, which are not conducive to the digestion of nutrients [22]. In our experiment, with the increase of the proportion of fermented feed, the content of fiber in the diet increased, resulting in the gradual decrease of digestibility. The lower nutrient digestibility of the 10% group may be due to the excessive growth of microorganisms caused by the high water content of the feed, which consumes the nutrients in the feed and reduces the nutrient concentration.

### Nutrient metabolic rate

Feed fermentation can improve the nutrient metabolism of poultry. It was found that fermented feed could increase the expression of AMY2A and CCK in pancreas of broilers and increase the secretion of amylase and cholecystokinin in pancreas (SUn) [23]. Al-Khalaifah et al [24] found that fermented dry beer grain (DBG) can promote the expression of genes related to digestion and nutrient transport more than enzyme treated DBG, and these genes can regulate the nutrient utilization required by poultry growth. Lawal et al [25] reported that broilers fed diets supplemented with fermented palm kernel had higher apparent digestibility of DM, N, and ash than those in the control. Ahmed et al [26] also obtained similar results. The digestibility of CP in diets containing fermented rapeseed meal was higher than that in broilers without fermented rapeseed meal.

Nutrient digestibility is a quantitative assessment of nutritional and physiological phenomena related to digestive capacity and intestinal function. In our experiment, the apparent metabolic rates of EE and Ca of broilers were increased after adding fermented material. Lactic acid produced by fermentation can decreased the pH of feed, and then reduce the pH of digestive tract, which provides a good acidic environment for Ca absorption. The apparent metabolic rate of CP was increased by adding 20% fermented feed. This may be because the beneficial microorganisms in the fermentation broth can produce some metabolites, such as lactic acid, bacteriocin, antibacterial substances, higher alcohols, etc., which can reduce the pH of digestive tract, inhibit, or kill harmful bacteria such as *Escherichia coli*, and improve the digestion and absorption capacity of intestines [25,27]. In this experiment, with the increase of the proportion of fermented feed, the apparent digestibility of nutrients increased first and then decreased. It is suggested that the proportion of fermented feed in animal feed should be further explored.

### Cecal microflora structure

Intestinal microflora have positive effects on the host in many ways, such as regulating intestinal motility and immune homeostasis, promoting nutrient absorption [28], producing vitamins, metabolizing bile acids and sterols [29]. In addition, intestinal flora can also protect the host from pathogens [28] and inflammatory intestinal diseases, thus improving intestinal health. Generally, intestinal flora is affected by many factors, such as diet, age, host genotype, pathogen infection and feed additives. In our experiment, the microbial abundance index and Shannon index in cecum of broilers fed with fermented feed showed an increasing trend compared with the control, and the highest was the 10% WFF and 10% DFF, but there was no statistical difference.

At the phylum level, we found that the cecal microflora of all experimental groups were dominated by *Bacteroides* and *Firmicutes*, which was consistent with the observation of previous studies [30–33]. The abundance of *Firmicutes* is often positively correlated with the growth performance of animals [34,35]. At genus level, the abundance of *Bacteroides*, *Clostridium xiva*, and *Clostridium IV* in the cecum of broilers increased with the addition of fermented feed. Propionic acid produced by metabolism of pseudobacteroides can improve intestinal barrier function. In addition, *Bacteroides* have many genes required for polysaccharide metabolism [36,37]. In this experiment, the proportion of *Clostridium xiva* increased in all the treatment groups, while the proportion of *Clostridium IV* in 10% DFF and 25% DFF increased. The fermentation products of these two strains were mainly butyric acid. Butyric acid can stabilize the intestinal state, and provide energy to the body, so as to promote the growth of animals [38]. In conclusion, adding fermented feed can improve intestinal flora of broiler.

## CONCLUSION

Our results strongly indicated that the enzyme-bacteria co-fermented feed had a potential promoting effect on the growth performance and nutrient digestibility of broilers. In addition, the positive effects of enzyme-bacteria co-fermented feed on improving the intestinal microenvironment and optimizing the intestinal microflora structure were further confirmed in our study.

## Figures and Tables

**Table 1:** Basal diet composition and nutrient level of broilers (air dry basis, %)

Items	Content	

	1 to 21 d	22 to 42 d
Ingredients		
Corn	56.50	55.40
Soybean meal	25.55	22.20
Corn gluten meal	4.60	3.00
DDGS	3.00	3.00
Wheat bran	-	3.50
Soybean oil	1.00	3.50
Extruded soybean powder	5.00	5.00
Limestone	1.20	1.20
CaHPO_4_	1.80	1.80
NaCl	0.25	0.30
Choline chloride	0.10	0.10
Premix^1)^	1.00	1.00
Total	100.00	100.00
Nutrient content^2)^		
ME (MJ/kg)	12.42	12.78
CP	20.77	18.53
DM	87.36	87.38
EE	4.45	6.91
Ca	1.08	0.96
Total P	0.68	0.68
Available P	0.45	0.42
Lysine	1.11	0.98
Methionine	0.48	0.44
Threonine	0.73	0.65
Tryptophan	0.19	0.18

DDGS, dried distillers grains with solubles; ME, metabolizable energy; CP, crude protein; DM, dry matter; EE, ether extract.

1) Premix provided nutrients value of diet (/kg): Cu 25 mg, I 1.0 mg, Fe 100 mg, Mn 120 mg, Se 0.15 mg, Zn 80 mg; vitamin A 18,000 IU, vitamin D_3_ 2,800 IU, vitamin E ≥90 mg, vitamin K_3_ ≥7.2 mg, vitamin B_1_ ≥6.84 mg, vitamin B_2_ ≥27 mg, vitamin B_6_ ≥13.5 mg, vitamin B_12_ ≥0.108 mg, nicotinamide ≥108 mg, calcium pantothenate ≥45 mg, folic acid ≥19.8 mg, biotin ≥0.72 mg.

2) Proximate nutrients are measured values, other nutrients are calculated values.

**Table 2:** Composition of enzyme-bacteria co-fermented feed (air-dry basis, %)

Ingredients	1 to 21 days	22 to 42 days
Corn	63.02	63.61
Soybean meal	28.51	25.49
Corn gluten meal	5.13	3.44
DDGS	3.34	3.44
Wheat bran	-	4.02
Total	100.00	100.00

DDGS, dried distillers grains with solubles.

**Table 3:** Reaction system for first polymerase chain reaction

Ingredients	Volume
2×Hieff Robust PCR Master Mix	15 μL
Bar-PCR primer F	1 μL
Primer R	1 μL
PCR product	10 to 20 ng
H_2_O	9 to 12 μL
Total volume	30 μL

PCR, polymerase chain reaction.

**Table 4:** First round polymerase chain reaction amplification reaction procedure

Reaction temperature (°C)	Reaction time	
94	3 min	
94	30 s	5 cycles
45	20 s	
65	30 s	20 cycles
94	20 s	
55	20 s	
72	30 s	
72	5 min	

**Table 5:** Reaction system for second polymerase chain reaction

Ingredients	Volume
2×Hieff Robust PCR Master Mix	15 μL
Primer F	1 μL
Index-PCR Primer R	1 μL
PCR products	20 to 30 ng
H_2_O	9 to 12 μL
Total volume	30 μL

PCR, polymerase chain reaction.

**Table 6:** Second round polymerase chain reaction amplification reaction procedure

Reaction temperature (°C)	Reaction time	
95	3min	
94	20s	5 cycles
55	20s	
72	30s	
72	5min	

**Table 7:** Feed composition changes after fermentation and drying (1–21 d/22–42 d, %)

Feed	Water	pH	Total acid	*Lactic acid bacteria* (CFU/g)
Pre-ferm.	12.65^a^/12.11^a^	6.16^a^/6.27^a^	0.67^a^/0.72^a^	(1.98×10^4^)^a^/(3.90×10^4^)^a^
After ferm.	31.23^b^/31.57^b^	4.14^b^/4.23^b^	2.28^b^/2.32^b^	(1.31×10^8^)^b^/(1.81×10^8^)^b^
Dry after ferm.	12.98^a^/12.55^a^	4.32^c^/4.21^b^	2.86^c^/3.13^c^	(3.57×10^6^)^a^/(2.77×10^6^)^a^
SEM	3.07/3.21	0.32/0.34	0.34/0.35	2.35×10^7^/3.05×10^7^
p-value	p<0.001/p<0.001	p<0.001/p<0.001	p<0.001/p<0.001	p<0.004/p<0.001

CFU, colony-forming unit; SEM, standard error of the mean.

a–c Values within a column with no or the same letter superscripts mean no significant difference (p>0.05); values with different small letter superscripts mean significant difference (p<0.05).

**Table 8:** Effect of the fermented feed on growth performance of broilers (g)

Items	Treatments^1)^						SEM	p-value

	BF	10% DFF	15% DFF	20% DFF	25% DFF	10% WFF		
1 to 21 days								
ADFI	43.33	43.57	43.87	43.37	43.87	45.08	0.25	0.341
ADG	31.1	32.22	32.22	31.7	31.14	32.12	0.18	0.2
F/G	1.39	1.35	1.36	1.37	1.41	1.41	0.01	0.257
Mortality/%	0	1.25	0	1.25	0	0	-	-
22 to 42 days								
ADFI	106.46^a^	111.76^b^	114.41^b^	107.35^a^	106.66^a^	111.69^b^	0.68	<0.001
ADG	62.67^a^	66.56^b^	68.18^b^	65.43^ab^	62.84^a^	67.56^b^	0.49	<0.001
F/G	1.7	1.68	1.68	1.64	1.7	1.66	0.01	0.452
Mortality/%	1.25	0	0	2.5	1.25	3.75	-	-
1 to 42 days								
ADFI	74.06^a^	77.67^b^	79.14^b^	74.10^a^	74.70^a^	78.45^b^	0.49	<0.001
ADG	47.23^a^	50.51^b^	51.39^b^	49.49^ab^	47.91^ab^	50.74^b^	0.31	<0.001
F/G	1.57	1.54	1.54	1.5	1.56	1.54	0.02	0.139
Mortality/%	1.25	1.25	0	3.75	1.25	3.75	-	-

SEM, standard error of the mean; ADFI, average daily feed intake; ADG, average daily gain; F/G, average daily feed intake/average daily gain.

1) BF, basal diet; DFF, dried fermented feed; WFF, wet fermented feed.

a,b Values within a row with no or the same letter superscripts mean no significant difference (p>0.05); values with different small letter superscripts mean significant difference (p<0.05).

**Table 9:** Effect of the fermented feed on nutrient metabolic rate of broilers (%)

Items	Treatments^1)^						SEM	p-value

	BF	10% DFF	15% DFF	20% DFF	25% DFF	10% WFF		
DM	69.07	72.35	71.01	71.8	70.88	69.3	0.58	0.533
CP	49.55	55.64	54.87	56.64	54.7	54.6	0.91	0.295
EE	71.44	81.44	77.65	77.5	76.68	78.44	0.94	0.06
Ca	37.5	40.63	48.49	42.64	41.21	39.73	0.19	0.138
P	36.61	41.15	38.8	40.95	40.05	41.73	1.16	0.412
Energy	73.62	76.73	75.76	74.71	73.91	73.66	0.59	0.598

SEM, standard error of the mean; DM, dry matter; CP, crude protein; EE, ether extract.

1) BF, basal diet; DFF, dried fermented feed; WFF, wet fermented feed.

Values within a column with no letter superscripts mean no significant difference (p>0.05).

**Table 10:** Effect of the fermented feed on proportion of bacteria at phylum level in broilers (%)

Items	Treatments^1)^						SEM	p-value

	BF	10% DFF	15% DFF	20% DFF	25% DFF	10% WFF		
Firmicutes	63.23	59.67	76.44	68.31	70	70.7	2.11	0.26
Bacteroidetes	32.05	31.85	13.73	21.87	23.77	26.38	2.41	0.238
Proteobacteria	2.57	6.85	6.25	7.9	2.69	1.63	0.98	0.296
Synergistetes	0.87^ab^	0.52^ab^	2.02^a^	0.97^b^	1.46^ab^	0.34^b^	0.17	0.035
Unclassified	0.81	0.83	1.32	0.75	0.8	0.59	0.08	0.122
Actinobacteria	0.17	0.14	0.14	0.13	0.23	0.15	0.02	0.642
Euryarchaeota	0.24^a^	0.04^b^	0.05^b^	0.02^b^	0.06^b^	0.13^ab^	0.02	0.035
Others	0.06	0.09	0.05	0.06	0.1	0.09	0.15	0.408

SEM, standard error of the mean.

1) BF, basal diet; DFF, dried fermented feed; WFF, wet fermented feed.

a,b Values within a row with no or the same letter superscripts mean no significant difference (p>0.05); values with different small letter superscripts mean significant difference (p<0.05).

**Table 11:** Effect of the fermented feed on proportion of bacteria at genus level in broilers (%)

Items	Treatments^1)^						SEM	p-value

	BF	10% DFF	15% DFF	20% DFF	25% DFF	10% WFF		
Unclassified	38.15	37.68	48.45	35.97	47.17	40.11	2.09	0.396
*Alistipes*	24.64	21.76	9.03	14.69	12.52	17.18	1.75	0.090
*Ruminococcus*	3.86^b^	6.24^ab^	2.59^b^	3.79^b^	3.71^b^	8.58^a^	0.53	0.008
*Lactobacillus*	3.65	1.84	9.88	6.02	2.19	2.37	0.94	0.092
*Faecalibacterium*	4.44	4.19	2.91	7.77	2.83	3.40	0.78	0.471
*Bacteroides*	2.04	4.34	1.39	2.35	6.20	2.99	0.51	0.063
*Subdoligranulum*	3.07	1.06	5.43	1.97	1.27	3.84	0.57	0.202
*Romboutsia*	1.69	2.62	3.04	3.08	1.72	0.62	0.58	0.820
*Barnesiella*	1.97	1.42	1.87	1.56	1.73	3.27	0.40	0.824
*Clostridium XlVa*	1.17	1.54	1.22	2.14	2.72	2.14	0.24	0.401
*Clostridium IV*	1.47	1.82	1.42	1.11	2.03	1.24	0.11	0.105
*Bilophila*	0.59	0.52	2.51	4.14	0.40	0.70	0.60	0.378
*Butyricicoccus*	1.55	0.87	0.89	0.85	1.69	1.98	0.20	0.438
*Vampirovibrio*	1.02	1.89	1.90	0.66	0.93	0.46	0.29	0.596
*Dorea*	0.99	1.68	0.54	1.02	0.94	1.16	0.17	0.577

SEM, standard error of the mean.

1) BF, basal diet; DFF, dried fermented feed; WFF, wet fermented feed.

a,b Values within a row with no or the same letter superscripts mean no significant difference (p>0.05); values with different small letter superscripts mean significant difference (p<0.05).

**Table 12:** Effect of the fermented feed on cecal microbial alpha diversity in broilers

Items	Treatments^1)^						SEM	p-value

	BF	10% DFF	15% DFF	20% DFF	25% DFF	10% WFF		
ACE	4,481.98	4,826.40	4,347.21	4,709.64	4,737.21	4,854.52	160.29	0.941
Chao 1	3,081.73	3,338.83	2,959.54	3,253.63	3,275.95	3,392.54	101.24	0.838
Shannon	4.26	4.37	4.1	4.35	4.65	4.38	0.07	0.277
Simpson	0.07	0.06	0.07	0.06	0.03	0.05	0.01	0.395
Coverage	0.99	0.99	0.99	0.99	0.99	0.99	0.01	0.175

SEM, standard error of the mean; ACE, the ACE estimator (http://www.mothur.org/wiki/Ace); Chao 1, the Chao 1 estimator (http://www.mothur.org/wiki/Chao).

1) BF, basal diet; DFF, dried fermented feed; WFF, wet fermented feed.

Data within a column with no letter superscripts mean no significant difference (p>0.05).
